# Editorial: Navigating the nexus of big data, AI, and public health: transformations, triumphs, and trials in multiple sclerosis care access

**DOI:** 10.3389/fdata.2025.1682151

**Published:** 2025-10-01

**Authors:** Immanuel Azaad Moonesar, M. V. Manoj Kumar, Khulood Alsayegh, Ayat Abu-Agla, Likewin Thomas

**Affiliations:** ^1^Mohammed Bin Rashid School of Government, Dubai, United Arab Emirates; ^2^International Vaccine Institute, Seoul, Republic of Korea; ^3^NITTE Meenakshi Institute of Technology, Bengaluru, India; ^4^Dubai Health Authority, Dubai, United Arab Emirates; ^5^University of Birmingham Dubai, Dubai, United Arab Emirates

**Keywords:** artificial intelligence (AI), big data, healthcare accessibility, geographic disparities, Multiple Sclerosis, Disease-Modifying Therapies, health equity, geospatial analysis

## The convergence revolution in health and society

In today's data-driven world, the confluence of big data, artificial intelligence (AI), and public health emerges as a pivotal juncture poised to reshape healthcare paradigms. This Research Topic brings together four groundbreaking studies that demonstrate how the integration of advanced analytics, machine learning, and vast datasets is transforming our approach to public health challenges, with particular emphasis on addressing geographic and socioeconomic disparities in healthcare access—exemplified by the critical need for equitable access to Disease-Modifying Therapies for Multiple Sclerosis patients.

The collection of articles in this Research Topic exemplifies the transformative potential of big data and AI in addressing healthcare accessibility challenges that disproportionately affect vulnerable populations. From educational accessibility during global crises to gender bias in healthcare AI systems, these contributions illuminate the complex barriers that must be overcome to ensure equitable access to specialized treatments and healthcare services.

## Educational equity and public health: lessons from the pandemic

Santos et al. present a compelling analysis of how the COVID-19 pandemic exacerbated educational inequalities in Brazil's Pará state, using Bayesian networks to examine the intersection of socioeconomic factors, technology access, and academic performance. The research employed a mixed-methods approach, analyzing quantitative data from ENEM results (2020–2022) and qualitative interviews with educators and students. Their findings reveal critical insights into how public health crises disproportionately affect vulnerable populations—paralleling the challenges faced by MS patients in accessing specialized care during disrupted healthcare delivery.

The highest dropout rates were recorded among students with a family income of up to one minimum wage, highlighting the barriers posed by limited access to technology and infrastructure for remote learning. This research demonstrates the power of probabilistic modeling in understanding complex social determinants of health and education, with Santos et al. showing how higher maternal employment and education levels correlated with improved student performance, illustrating the interconnected nature of social determinants that extend to healthcare accessibility patterns.

## Addressing gender bias in healthcare AI systems

Joshi provides a critical examination of how big data and AI technologies can both advance and hinder gender equality in healthcare access and treatment. This perspective article addresses one of the most pressing ethical challenges in contemporary healthcare technology: bias is a big challenge when implementing AI systems in medical settings, particularly relevant for conditions like Multiple Sclerosis, where gender disparities in diagnosis and treatment access are well-documented.

The contribution by Joshi highlights three critical categories where machine learning can facilitate accessible, affordable, personalized, and evidence-based healthcare for women, while simultaneously addressing the algorithmic bias that threatens to undermine these advances. This work exemplifies the critical need for intersectional approaches to healthcare AI development that consider both the transformative potential and the ethical implications of these technologies in specialized treatment contexts such as neurological care.

## Social media analytics for public health insights

Chen et al. (2024) contribute valuable insights into the practical challenges of leveraging social media data for public health research and healthcare accessibility analysis. Social media has profoundly changed our modes of self-expression, communication, and participation in public discourse, generating volumes of conversations and content that cover every aspect of our social lives. Their research addresses the critical gap between the theoretical potential of social media analytics and the practical hurdles researchers face in accessing and utilizing these data sources for understanding patient experiences and healthcare navigation challenges.

The work by Chen et al. (2024) is particularly relevant for understanding patient perspectives on healthcare accessibility, treatment barriers, and health-seeking behaviors—essential components for developing comprehensive models of healthcare access, such as those needed for MS treatment planning as an example of the United Arab Emirates. Social media platforms provide unprecedented access to real-time patient sentiment, treatment experiences, and geographic variation in healthcare access that can inform policy and practice improvements.

## Knowledge-based recommendation systems in healthcare contexts

The fourth contribution explores advanced techniques in knowledge-based recommendation systems, with direct relevance to clinical decision support and personalized treatment recommendations for complex conditions like Multiple Sclerosis. This research demonstrates how sophisticated recommendation algorithms can be applied to support clinical decision-making in specialized care contexts, patient education about treatment options, and healthcare resource allocation—all critical components for ensuring equitable access to Disease-Modifying Therapies.

The integration of knowledge-based systems with machine learning approaches represents a significant advancement in creating transparent, including clinically interpretable, explainable AI tools for specialized healthcare settings. This work addresses the crucial challenge of AI interpretability in neurological care contexts, where understanding the rationale behind treatment recommendations is essential for both clinical acceptance and patient adherence to complex therapeutic regimens.

## Cross-cutting themes: geographic equity and specialized care access

When handling private health information, strong protections are needed to prevent breaches and unauthorized use. Across all four articles, several critical themes emerge that are directly applicable to addressing geographic and socioeconomic disparities in specialized healthcare access, particularly relevant for conditions requiring ongoing Disease-Modifying Therapy.

### Geospatial analysis and access mapping

The methodological approaches demonstrated across these studies provide frameworks for mapping healthcare accessibility patterns. Santos et al.'s geographic analysis of educational access barriers during the pandemic offers direct parallels to understanding how distance, infrastructure, and socioeconomic factors create barriers to specialized medical care in diverse geographic regions, including accessibility heatmaps or location allocation models, which are used in health system planning to reduce spatial inequality.

### Algorithmic bias and treatment equity

Joshi's examination of gender bias in healthcare AI systems highlights challenges that extend to all aspects of specialized care delivery. The research demonstrates how historical inequities in healthcare access can be embedded in algorithmic systems used for treatment allocation, facility planning, diverse training data and patient risk stratification—directly relevant to ensuring equitable DMT access across different populations.

### Mixed-methods healthcare research

The integration of quantitative analytics with qualitative patient experience data, demonstrated by Santos et al. and supported by Chen et al.'s (2024) social media analysis framework, provides essential methodological guidance for comprehensive healthcare accessibility studies that combine geospatial analysis with patient journey mapping.

## Implementation science for healthcare equity

The research presented in this Research Topic moves beyond theoretical accessibility models to address practical implementation challenges in healthcare delivery. Chen et al.'s (2024) analysis of data collection hurdles provides essential guidance for researchers conducting patient experience studies, while Santos et al.'s socioeconomic analysis offers actionable insights for policymakers addressing geographic healthcare disparities.

These studies demonstrate that successful implementation of equitable healthcare access requires not only advanced analytical capabilities but also careful attention to privacy protection, stakeholder engagement, and systematic approaches to addressing the social determinants that influence treatment access and adherence patterns.

## Future directions for healthcare accessibility research

Policymakers can use big data to subsequently review the social factors, among others, behind these health disparities. The collective insights from these four articles point toward several critical areas for future research in healthcare accessibility, with direct applications to specialized treatment access challenges.

The development of comprehensive accessibility indices that integrate geographic, socioeconomic, and system-level factors becomes increasingly crucial for conditions requiring complex, ongoing treatment regimens. The methodological frameworks demonstrated in this Research Topic provide essential building blocks for creating predictive models that can identify patients at risk of treatment discontinuation and guide targeted intervention strategies.

Furthermore, the research highlights the importance of real-time monitoring systems that can track accessibility patterns and identify emerging barriers to care. The integration of social media analytics, demonstrated by Chen et al. (2024), with geospatial analysis approaches offers promising directions for developing systems that monitor healthcare accessibility in real time.

## Conclusion: toward equitable specialized care

This Research Topic serves as both a demonstration of current analytical capabilities and a roadmap for addressing persistent challenges in healthcare accessibility and equity. The contributing articles establish important methodological precedents for conducting comprehensive accessibility studies while emphasizing the continued need for innovation in addressing geographic and socioeconomic barriers to specialized care.

As healthcare systems worldwide grapple with ensuring equitable access to specialized treatments like Disease-Modifying Therapies for Multiple Sclerosis, the approaches demonstrated in this Research Topic offer essential tools for understanding, measuring, and addressing accessibility challenges. The intersection of advanced analytics with social determinants research represents a critical pathway toward more equitable healthcare delivery systems.

The ultimate goal—ensuring that all patients have access to life-changing treatments regardless of their geographic location or socioeconomic status—requires continued investment in both analytical capabilities and the implementation science necessary to translate insights into improved healthcare delivery. The work presented in this Research Topic represents significant progress toward this goal while highlighting the ongoing need for comprehensive, multidisciplinary approaches to addressing healthcare equity challenges.
